# Multi‐omic molecular profiling guide’s efficacious treatment selection in refractory metastatic breast cancer: a prospective phase II clinical trial

**DOI:** 10.1002/1878-0261.13091

**Published:** 2021-09-12

**Authors:** Mariaelena Pierobon, Nicholas J. Robert, Donald W. Northfelt, Mohammad Jahanzeb, Shukmei Wong, Kimberly A. Hodge, Elisa Baldelli, Jessica Aldrich, David W. Craig, Lance A. Liotta, Sanja Avramovic, Janusz Wojtusiak, Farrokh Alemi, Julia D. Wulfkuhle, Angela Bellos, Rosa I. Gallagher, David Arguello, Amber Conrad, Ariane Kemkes, David M. Loesch, Linda Vocila, Bryant Dunetz, John D. Carpten, Emanuel F. Petricoin, Stephen P. Anthony

**Affiliations:** ^1^ George Mason University Manassas VA USA; ^2^ US Oncology Network/Virginia Cancer Specialists Fairfax VA USA; ^3^ Mayo Clinic Arizona Scottsdale AZ USA; ^4^ A Division of 21^st^ Century Oncology Florida Precision Oncology Raton FL USA; ^5^ Translational Genomics Research Institute Phoenix AZ USA; ^6^ Department of Health Administration and Policy George Mason University Fairfax VA USA; ^7^ Caris Life Sciences Phoenix AZ USA; ^8^ Paradigm Diagnostics Phoenix AZ USA; ^9^ Translational Drug Development (TD2) Scottsdale AZ USA; ^10^ The Side‐Out Foundation Fairfax VA USA; ^11^ Evergreen Hematology‐Oncology Spokane WA USA

**Keywords:** metastatic breast cancer, multi‐omic molecular profiling, precision medicine, relational database, TOPO1 inhibitors

## Abstract

This prospective phase II clinical trial (Side Out 2) explored the clinical benefits of treatment selection informed by multi‐omic molecular profiling (MoMP) in refractory metastatic breast cancers (MBCs). Core needle biopsies were collected from 32 patients with MBC at trial enrollment. Patients had received an average of 3.94 previous lines of treatment in the metastatic setting before enrollment in this study. Samples underwent MoMP, including exome sequencing, RNA sequencing (RNA‐Seq), immunohistochemistry, and quantitative protein pathway activation mapping by Reverse Phase Protein Microarray (RPPA). Clinical benefit was assessed using the previously published growth modulation index (GMI) under the hypothesis that MoMP‐selected therapy would warrant further investigation for GMI ≥ 1.3 in ≥ 35% of the patients. Of the 32 patients enrolled, 29 received treatment based on their MoMP and 25 met the follow‐up criteria established by the trial protocol. Molecular information was delivered to the tumor board in a median time frame of 14 days (11–22 days), and targetable alterations for commercially available agents were found in 23/25 patients (92%). Of the 25 patients, 14 (56%) reached GMI ≥ 1.3. A high level of DNA topoisomerase I (TOPO1) led to the selection of irinotecan‐based treatments in 48% (12/25) of the patients. A pooled analysis suggested clinical benefit in patients with high TOPO1 expression receiving irinotecan‐based regimens (GMI ≥ 1.3 in 66.7% of cases). These results confirmed previous observations that MoMP increases the frequency of identifiable actionable alterations (92% of patients). The MoMP proposed allows the identification of biomarkers that are frequently expressed in MBCs and the evaluation of their role as predictors of response to commercially available agents. Lastly, this study confirmed the role of MoMP for informing treatment selection in refractory MBC patients: more than half of the enrolled patients reached a GMI ≥ 1.3 even after multiple lines of previous therapies for metastatic disease.

## Graphical abstract

This prospective trial assessed the role of multi‐omic molecular profiling in informing treatment selection for metastatic breast cancer patients. Molecular profiles were collected in 14 business days and targetable alterations for commercial agents were identified in 92% of patients; 56% of patients reached a growth modulation index ≥ 1.3 after multiple lines of previous therapies. This work confirms the unique role for multi‐omic molecular profiling in guiding treatment selection for metastatic patients.

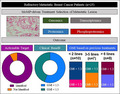

**Abbreviations**
AR: androgen receptor
CISH: chromogenic in situ hybridization
GMI: growth modulation index
IHC: immunohistochemistry
LCM: laser capture microdissection
MBC: metastatic breast cancers
MoMP: multi‐omic molecular profiling
PFS: progression‐free survival
RPPA: reverse phase protein microarray
SNV: single nucleotide variation
TGen: Translational Genomics Research Institute
TSC: treatment selection committee
TTP: time to progression

## 1 Introduction

While breast cancer mortality rates have declined over the last two decades and overall survival rates for patients diagnosed with early stage tumors are relatively favorable, the development of distant metastases still remains the main cause of cancer‐related death in this group of patients [1]. Although dozens of compounds, including conventional chemotherapeutic and targeted agents, have been granted federal approval as treatment options for breast cancer patients, gold standards for metastatic breast cancer (MBC) still remain poorly defined. This pilot study assessed the role of multi‐omic molecular profiling (MoMP) in identifying actionable therapeutic targets that can successfully guide treatment selection for commercially available agents in patients with refractory MBC after multiple lines of therapy.

The introduction of molecular profiling has led to a fundamental paradigm shift in the understanding of breast cancer biology and disease management [2, 3, 4]. Biomarker‐defined molecular profiles are becoming an integral part of the therapeutic decision‐making process, with NGS‐based analyses playing a primary role in the vast majority of precision oncology initiatives. However, a number of previous studies have shown that genomic analyses alone can identify targetable alterations in only a small to moderate proportion of cancer patients and this approach fails to capture tumors’ genomic‐independent mechanisms of adaptation in a host microenvironment [5, 6, 7].

Previous work has suggested that MoMP of the metastatic lesion provides molecular insights for tailoring treatment in a high proportion of patients and can be highly beneficial in the metastatic setting [8, 9, 10, 11, 12]. Building upon the Side Out 1 trial experience [13], this study confirmed the exceptional role of MoMP in identifying functionally deranged drug targets and chemo‐predictive markers in MBC. This work also provided further evidence of the clinical utility of MoMP in patients whose tumors have progressed over numerous lines of therapy. In addition, the approach proposed allowed us to identify genomic, proteomic, and phosphoproteomic actionable targets frequently expressed in MBC and assess their role as predictors of response to on‐label and off‐label FDA‐approved agents. Finally, broad scale molecular data generated through this work were used to develop a novel publicly available relational database where clinical, pathological, molecular, and outcome data can be easily accessed by the research and medical community.

## 2 Methods

### 2.1 Trial design

This open‐label, multicenter pilot study assessed the role and impact of MoMP for treatment selection by a molecular tumor board in highly pretreated MBC patients with a PFS interval < 6 months. Patients were enrolled in the Side Out 2 trial (ClinicalTrial.gov Identifier: NCT01919749) at four US institutions including two universities/research centers and two community‐based cancer centers. The study protocol was approved by independent Institutional Review Boards, and the trial was conducted in accordance with the principles of Code of Federal Regulations, and the Declaration of Helsinki. All patients provided voluntary written informed consent before undergoing study‐related procedures.

The primary objective of the study was to explore whether treatment with FDA‐approved anti‐cancer agents based on MoMP provides clinical benefits superior to empiric treatment selection in patients affected by refractory MBC. Secondary objectives of the analysis were as follows: a) to determine the frequency by which MoMP identified target(s) for FDA‐approved agents, b) to assess the percent of time in which MoMP‐based treatment selection differed from empirical physician choice, c) to identify tissue biomarker(s) with high predictive value of response in MBC, and d) to assess RECIST 1.1 based response rate and percentage of patients without progression at 4 months from treatment initiation.

**Fig. 1:**
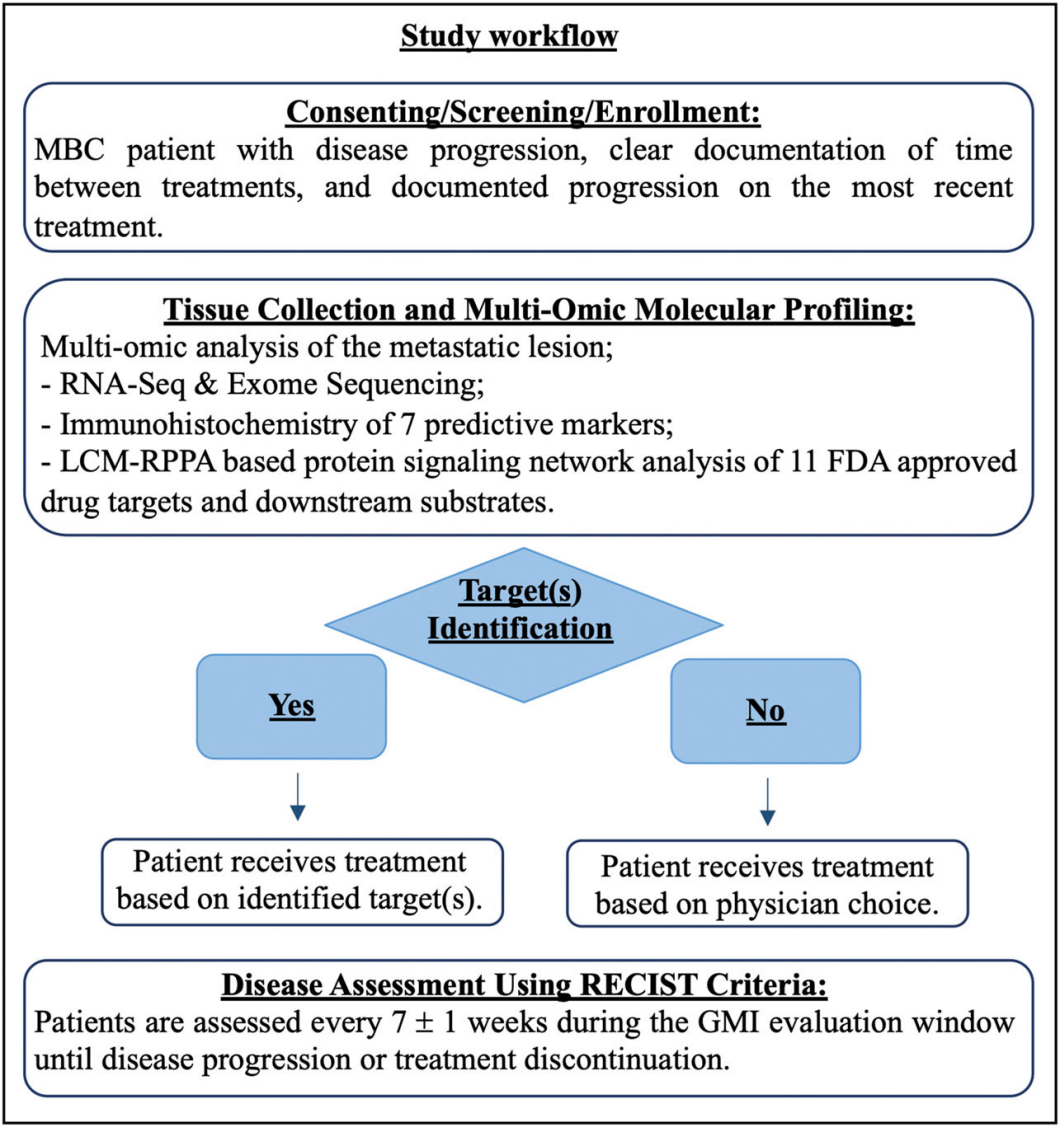
Schematic Workflow describing screening and enrollment procedure, MoMP collection, and treatment selection. After patients were enrolled in the study, a biopsy of the metastatic lesion was collected and sent for MoMP. Molecular data were discussed by the TSC and used to identify FDA‐approved agents targeting the identified targets.

Patients with documented refractory breast cancer after at least one prior chemotherapeutic and/or hormonal regimen for metastatic disease and a PFS of less than 6 months were eligible for the study. Eligibility was verified by the principal investigator (SPA) (Table S1). Four 18‐gauge core needle biopsies of a metastatic lesion were collected from each patient and used to generate a comprehensive MoMP (Fig. 1). ER, PR, and HER2 status were assessed on the metastatic lesions using immunohistochemistry (IHC) (ER, PR) or chromogenic in situ hybridization (CISH) for HER2 status as part of the molecular profile (Table 1). Molecular information was reviewed by the Treatment Selection Committee (TSC), a tumor board led by the principal investigator (SPA) and comprised of clinical investigators/treating physicians (DWN/MJ/NJR/SPA), laboratory investigators (AC/DA/DML/EFP/JDC/LAL/MP), and independent representatives (LV). Treatment selection was based on biologically relevant elements identified by MoMP along with patients’ clinical and prior therapeutic histories. Treatment was selected using FDA‐approved off‐label or on‐label agents either in monotherapy or in combination for approved oncology treatments as contemplated by the study protocol. In the event that MoMP did not identify any molecular target, patients received the treatment selected on an empirical basis by the treating physician within the context of the TSC discussion. All treatments were administered according to manufacturer’s instructions and standard institutional practice.

**Table 1:** Characteristics of the 25 patients that were treated based on their MoMP. Listed are patients’ GMI, receptor status of the metastatic lesion, metastatic site, targets used for treatment selection, and MoMP‐based selected treatment.

Subject ID	GMI		Previous therapies	Receptor Status*	Target lesion	MoMP Targets	MoMP‐based Treatment
02‐027	0.3 0.4	8		ER+/PR‐/HER2‐	Omentum	AR; ER; TOPO1	Megestrol Acetate (1st line); Irinotecan (2nd line)
02‐012	0.4 1.3	7		ER+/PR+/HER2‐	Liver	AR; ER; TOPO1; TS	Capecitabine; Irinotecan (1st line); Megestrol Acetate (2nd line)
02‐037	0.5	4		ER+/PR+/HER2‐	Liver	TOPO1	Irinotecan
02‐043	0.5	7		ER+/PR‐/HER2‐	Liver	No biomarker(s)	Eribulin
02‐008	0.5	7		ER+/PR+/HER2‐	Chest wall/Skin	ER; p‐p70S6K	Everolimus; Exemestane
02‐006	0.6	1		ER+/PR‐/HER2‐	Lymph node	p‐AKT; p‐HER2; p‐HER3; p‐ERK; TS	Capecitabine; Lapatinib
02‐007**	0.7 1.0 1.9	3		ER+/PR‐/HER2‐	Chest wall/Skin	ER; p‐HER2; p‐ERK; TOPO1	Irinotecan (1st line); Lapatinib; Letrozole (2nd line); Eribulin (3rd line)
02‐021	0.8	2		ER+/PR‐/HER2‐	Omentum	ER; p‐p70S6K	Everolimus; Exemestane
02‐032	0.8	4		ER‐/PR‐/HER2‐	Chest wall/Skin	No biomarker(s)	Eribulin
02‐018	0.9	3		ER+/PR‐/HER2‐	Liver	TS, TYMP***	Capecitabine
02‐041	1.2	1		ER‐/PR‐/HER2‐	Chest wall/Skin	TOPO1	Irinotecan
02‐020	1.3	5		ER+/PR‐/HER2‐	Liver	ER; p‐p70S6K	Everolimus; Exemestane
02‐023****	1.3 1.7	1		ER‐/PR‐/HER2‐	Liver & Lymph node	EZH2****; Survivin****; TOPO1; TS; TUBB3****	Capecitabine; Irinotecan (1st line); Paclitaxel (2nd line)
02‐039	1.4	9		ER‐/PR‐/HER2+	Lung	TOPO1; HER2; p‐HER2; p‐ERK	Irinotecan; Trastuzumab
02‐014	1.4	7		ER+/PR‐/HER2‐	Lung	TOPO1	Irinotecan
02‐025	1.8	1		ER+/PR‐/HER2‐	Lymph node	TS	Capecitabine
02‐009	2.2 1.1	18		ER+/PR+/HER2‐	Abdominal mass	AR; ER; TS	Megestrol Acetate (1st line); Capecitabine; Vinorelbine (2nd line)
02‐003	2.4	1		ER+/PR‐/HER2‐	Liver	SPARC	Nab‐paclitaxel
02‐017	2.8	1		ER+/PR‐/HER2‐	Liver	TS; p‐EGFR; p‐HER2; p‐HER3; p‐ERK	Capecitabine; Lapatinib
02‐029*****	3.8	2		ER‐/PR‐/HER2‐	Chest wall/Skin	TOPO1	Irinotecan
02‐036	4.2	1		ER+/PR‐/HER2‐	Liver	TOPO1; TS	Capecitabine; Irinotecan
02‐010	6.1	5		ER+/PR+/HER2‐	Liver	TOPO1	Irinotecan
02‐019	7.2	9		ER‐/PR‐/HER2+	Chest wall/Skin	p‐EGFR; p‐HER2; p‐HER3; p‐ERK; HER2; HER3.	Docetaxel; Pertuzumab; Trastuzumab
02‐011	8.5	3		ER‐/PR‐/HER2‐	Liver	TOPO1; TS	Capecitabine; Irinotecan
02‐028	15.9	1		ER+/PR‐/HER2‐	Chest wall/Skin	TS	Capecitabine

* HER2 status was determined by CISH and ER and PR status by IHC.

** Metastatic lesion from a male breast tumor.

*** Thymidine Phosphorylase.

**** A second biopsy was collected from the same patient after recurrence.

***** HER2 status attributed based on whole exome sequencing analysis.

### 2.2 Laboratory assays

MoMP was performed by four different laboratories. Formalin fixed paraffin‐embedded (FFPE) tissues were used for: a) protein expression analysis and CISH for HER2 status measured under CLIA regulation by CARIS; b) targeted mutational analysis, chromosomal alterations, copy number variations, and mRNA and protein expression performed by Paradigm (now Exact Sciences) under CLIA regulation; and c) exome sequencing and RNA‐Seq analysis performed by the Translational Genomics Research Institute (TGen). Patient‐matched fresh frozen material was used by the Center for Applied Proteomics and Molecular Medicine at George Mason University for protein pathway activation mapping using laser capture microdissection (LCM) to isolate tumor epithelia followed by Reverse Phase Protein Microarray (RPPA) analysis as previously described [13]. The LCM‐RPPA assay, although not cleared nor approved by the FDA, has been developed and evaluated in accordance with the College of American Pathologist guidelines. Detailed description of the exome sequencing/RNA‐Seq analyses and RPPA has been previously reported [14]. A full list of measured analytes is provided in the Table S2.

### 2.3 Construction of the Side Out database

A novel open‐access database was created to capture demographic, clinical and pathological information, outcome data, and MoMP data from all MBC patients enrolled in the Side out 1 and 2 clinical trials [15]. The portal was created using the open‐source relational database management system MySQL and custom‐codes were written using the PHP server‐side scripting language. User interface, management, and authentication were created in WordPress. Higher level of security for the recorded information was achieved by using a secondary database along with custom‐codes during the data entry process. Data were entered following HIPAA regulation; fully de‐identified information were recorded and made available to users. Access to the database can be obtained at https://sideoutfoundation.gmu.edu/ upon request.

### 2.4 Outcome analysis and statistical considerations

Given the heavily pretreated nature of this cohort of patients (3.94 mean number of treatment for MBC), clinical outcomes were evaluated utilizing the Growth Modulation Index (GMI) as previously described [12]. While not conventionally used as primary end point of treatment efficacy, GMI has been previously used in phase II clinical trials to assess response in MBC patients [12]. In brief, the GMI was calculated as a ratio between progression‐free survival (PFS) on MoMP‐based treatment divided by the time to progression (TTP) on the previous treatment for metastatic disease. Because patients serve as their own control in the GMI calculation, this approach minimizes inter‐patient variability and increases statistical sensitivity [16]. A GMI of 1.3 was selected as a cut‐off value of treatment efficacy as a 30% improvement in PFS was previously defined of clinical significance [12, 13]. To meet the primary objective, at least 35% of participants had to achieve a GMI ≥ 1.3 in a sample population of 25 evaluable patients. Sample size was calculated using an exact single‐stage design for phase II studies with a one‐sided type I error of 5% and a power of 90% under the assumption that GMI ≥ 1.3 in ≤10% of patients would be clinically irrelevant, while a success rate ≥ 35% would merit further investigation.

Disease assessment was performed within a specified GMI window, defined as time between TTP on last prior treatment and 1.25 times the TTP. The specified GMI window was provided to the treating physician at time of enrollment to assure that a GMI ≥ 1.3 was not artificially obtained based on time of assessment. Patients were assessed every 7 ± 1 weeks during the GMI evaluation window until disease progression or treatment discontinuation. If progression was not observed at the end of GMI evaluation window, patients were assessed every 3 months until progression. For patients where molecular information was used to select more than one round of treatment, only the first GMI was included in the analysis (Table 1).

Continuous variables are reported as median and range while categorical variables are presented as frequencies and percentages. Mosaic plotting was performed in JMP v5.1 and modified in Photoshop v11 for publication purposes. Bar graphs were performed in GraphPad Prism v6. Molecular data were analyzed with R version 3.5.1, and data were visualized in the RStudio environment. The ComplexHeatmap package was used to generate heat maps.

## 3 Results

Between December 2013 and March 2016, a total of 32 metastatic breast patients with a PFS interval on last treatment of < 6 months were enrolled in the phase II Side Out 2 clinical trial. MoMP data were generated from core needle biopsies of a metastatic lesion collected from each patient at time of enrollment. Molecular profiles were performed by four different laboratories and included targeted IHC, CISH, targeted/exome sequencing and RNA‐Seq analysis, and protein pathway activation mapping of tumor epithelia by RPPA.

Of the 32 enrolled patients, 3 received standard of care; for the remaining 29, treatment selection was guided by MoMP. The median number of total prior therapies, including neoadjuvant and adjuvant lines, was 8.0 and median number of prior radiotherapy treatments was 2.0. At trial enrollment participants had received an average of 3.94 treatments (range 1–18) for their metastatic disease distributed as follows: ≤ 2 lines of treatment 15 patients, 3–5 previous treatments 9 patients, and ≥ 6 treatments 8 patients. Thus, this cohort mostly includes highly pretreated patients with aggressive disease (PFS on last treatment < 6 months).

**Fig. 2:**
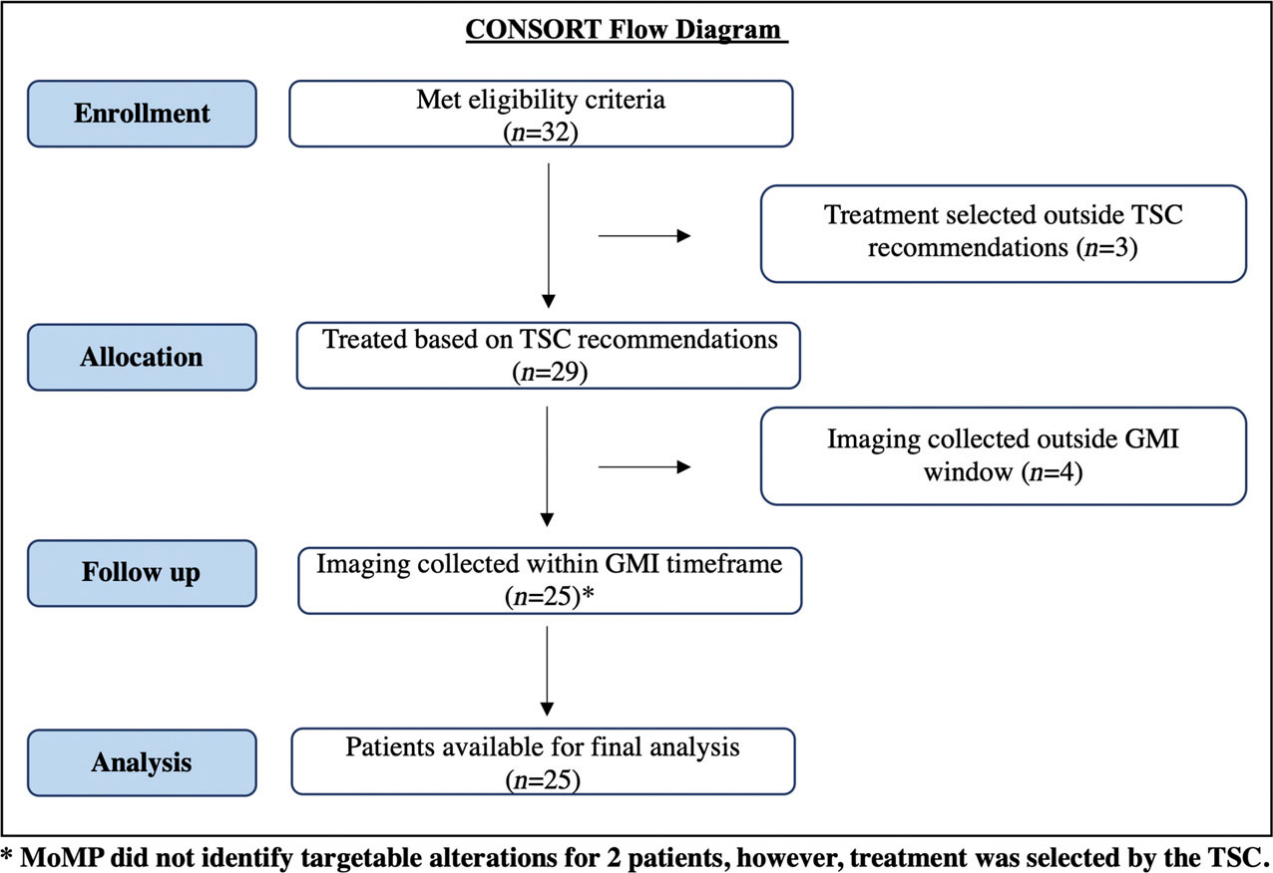
Modified CONSORT Flow Diagram describing the Side Out 2 trial enrollment process. The workflow captures the number of evaluable patients throughout the trial process including enrollment, patient allocation to treatment based on TSC recommendation, and follow‐up and analysis steps.

A total of 25 patients were evaluable within their GMI window, while follow‐up data for the remaining four patients were collected outside the GMI window and as such were not included in the final analysis (Fig. 2). Target lesion and ER/PR/HER2 status of the 25 eligible patients are described in Table 1. Briefly, 72% of patients were ER positive and 8% were HER2 positive based on MoMP collected on the metastatic lesion. Bone (71.9%), liver (46.9%), lung (43.8%), and lymph nodes (46.9%) were the most common sites of metastatic disease. All 25 patients were white of whom three were of Hispanic/Latino origin, including one male patient. Median age at trial enrollment was 62 (range 40–72).

### 3.1 Prospective biomarker analysis in metastatic breast cancer

IHC, RPPA, and mRNA data were collected prospectively by the three laboratories in a median timeframe of 14 days (range 11–22 days). Treatment was initiated on a median timeframe of 7 days (range 2–18) after MoMP data were discussed by a molecular tumor board or TSC. Exome sequencing data were delivered and discussed at a later time. IHC and RPPA data were successfully collected for all 25 patients. Exome sequencing data were generated for 22 patients, and mRNA expression data were available for 17 of the 25 patients. Patient characteristics, molecular information, clinical history, and outcome data are downloadable from the Side Out Foundation metastatic breast cancer portal at https://sideoutfoundation.gmu.edu.

**Fig. 3:**
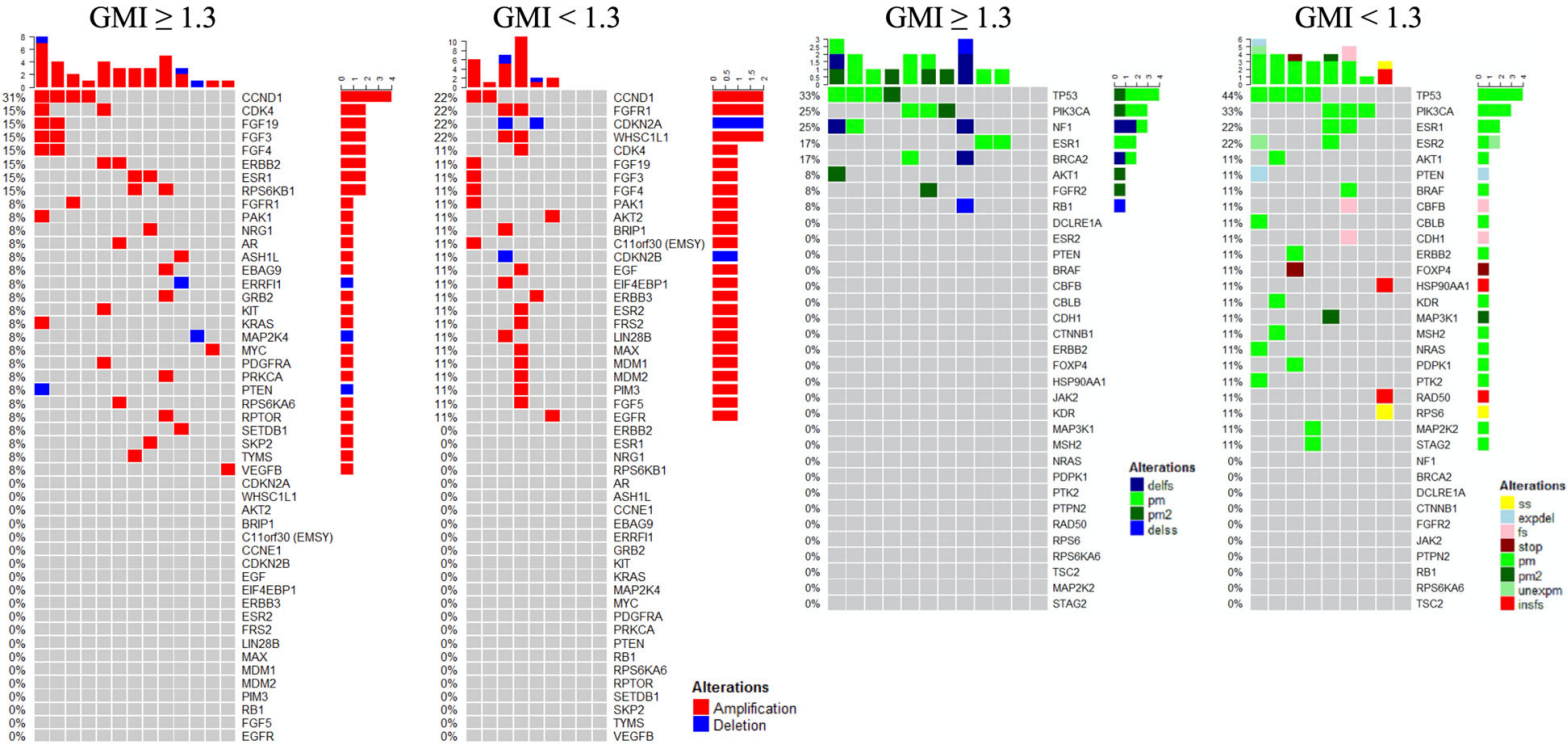
Frequencies of copy number variation and single nucleotide variation in the Side Out 2 trial based on patients’ GMIs. Heat map capturing NGS‐based single nucleotide variations for 22 of the 25 patients enrolled in the Side Out 2 trial; color‐codes reflect the type of alteration harbored by each patient. Legend delfs: deletion‐‐>frameshift; delss: deletion at splice site; expel: deletion at splice site that is expressed in RNA; fs: pm‐‐> frameshift; insfs: insertion‐‐>frameshift; pm: point mutation; pm2: 2‐point mutations; stop: premature stop; ss: splice site; unexpm: unexpressed pm.

**Fig. 4:**
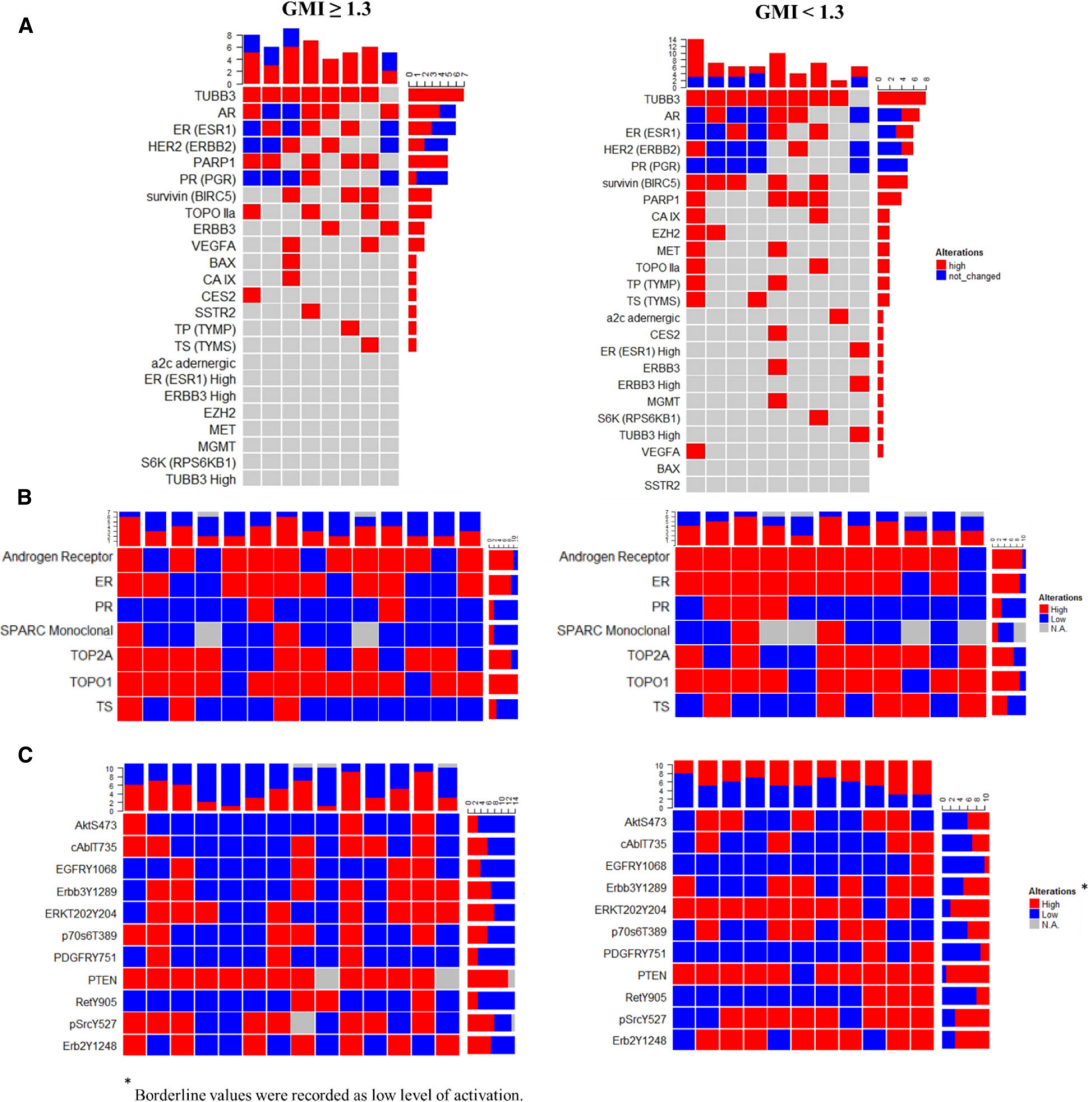
Summary of molecular findings in the Side Out 2 trial based on patients’ GMIs. Frequencies of gene expression, protein expression by immunohistochemistry and protein activation by RPPA (Panel A, Panel B, and Panel C, respectively) for 22 of the 25 patients enrolled in the Side Out 2 trial.

TP53 and PIK3CA Single Nucleotide Variations (SNVs) were among the most frequently detected genomic alterations in our cohort of patients. TP53 was mutated in 33% and 44% of responders and non‐responders, respectively (Fig. 3). Alterations of the PIK3CA were found in 25% of patients with GMI equal or greater of 1.3 and 33% in patients with GMI below 1.3. We have previously demonstrated that alterations of the PI3K/AKT signaling were more frequent in liver metastases compared to other metastatic sites [14]. Mutation of ESR1 was detected in 17% of responders and 22% of non‐responders (Fig. 3). Amplification of the CCND1 gene was also relatively frequent in this cohort of patients and it affected 31% and 22% of responders and non‐responders, respectively. Copy number variations of other cell cycle regulators (e.g., CDK4, CDKN2A) were also found in this cohort of patients, although at a lower frequency (Fig. 3). Gene expression analysis revealed increased expression levels of TUBB3 in both groups of patients (87.5% and 88.8% for responders and non‐responders, respectively) (Fig. 4A). Overexpression of Androgen Receptor (AR) and ESR1 was detected in at least a third of the patients. High levels of AR were also detected by IHC in 20 of the 25 patients (80%) including 10 of the 11 non‐responders (91%). Similarly, TOPO1 expression levels were elevated in 21 of the 25 patients (84.0%) (Fig. 4B). Seventeen of the 25 patients (68%) had low expression levels of thymidylate synthase (TS) by IHC. Finally, the RPPA data revealed increased ERK activity, measured as phosphorylation of the T202/Y204 residues, in 17 of the 25 patients including 8 (57.1%) responders and 9 non‐responders (81.8%) (Fig. 4C). Multiple members of the HER family were concomitantly activated in 8 of the 25 patients analyzed. This activation was not necessarily associated with HER2 amplification or overexpression.

### 3.2 Efficacy of MoMP‐based treatment selection in metastatic breast cancer

MoMP identified targetable biomarkers or predictive markers of response to on‐ and off‐label treatments for 23 of the 25 (92%) patients enrolled in the study (Table 1 and Table S3). Median time on treatment for this cohort of patients was 126 days (range 15–638). A total of 15 adverse events were recorded including 8 grade 1‐2 events, 5 grade 3, and 2 grade 4 events (Table S4). At 21 +/− 1 week, 13 patients remained on study with 15.4% demonstrating a partial response and 61.5% having stable disease. By 28 +/− 1 weeks, 9 patients remained on study with 22.2% of the patients demonstrating partial response and 33.3% stable disease. Thus, in a heavily pretreated patient population, with a PFS of < 6 months on the last therapy, a MoMP guided treatment selection seemed to provide some clinical benefit compared to prior empirically selected treatment. Of the 25 patients whose treatment selection was guided by MoMP, 56% (14/25) met the primary objective of the trial and reached a GMI ≥ 1.3 (Table 1 and Fig. S1). According to RECIST 1.1 criteria, best response was available for 24 of the 25 patients. Best response measured as PR and SD was detected in 12% (3/25) and 64% (16/25) of patients (Table S5).

High level of TOPO1 by IHC led to the selection of irinotecan‐based treatments in 48% (12/25) of the patients, with 5 patients receiving irinotecan as single agent. Capecitabine was the second most frequently selected treatment and was administered in combination (*n* = 6) or as single agent (*n* = 3) in 36% of cases. Selection of Capecitabine was based on MoMP and prior therapies given to a patient on a case‐by‐case basis. A total of 7 patients received an endocrine‐based therapy, 3 of whom were treated with the mTOR kinase inhibitor everolimus and exemestane. Selection of the combination treatment with everolimus and exemestane in ER+ patients was partially dictated by the presence of high level of phosphorylated p70S6 kinase, a direct downstream substrate and a read‐out of mTOR kinase activity. Finally, HER2 amplification and activation, measured as phosphorylated HER2 signature, led to the selection of an HER2 targeted agent in 5 patients. Of interest, for one patient (case N02‐017), the anti‐HER2 targeted agent lapatinib was selected in combination with capecitabine based on the activation level of multiple HER family members (including EGFR, HER2, and HER3) and the downstream substrate ERK. While the primary tumor of this patient was an HER2‐positive lesion, the metastatic specimen collected for the trial did not harbor an HER2 amplification. The patient reached a GMI of 2.8 while on trial.

**Fig. 5:**
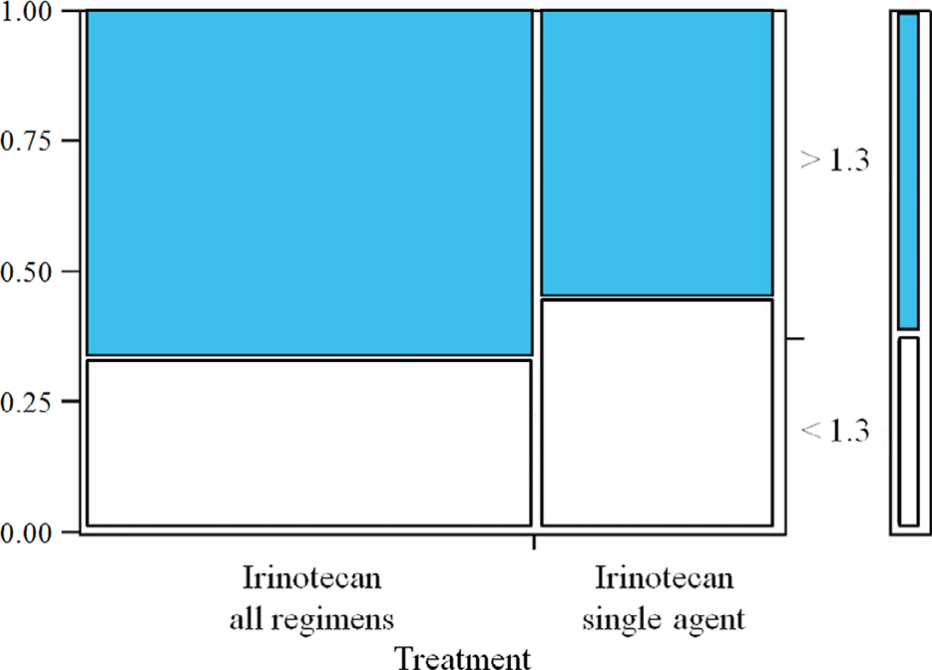
Mosaic plot displaying GMI in patients with high TOPO1 expression treated with irinotecan. IHC‐based frequencies of TOPO1 expression (left axis) along with GMI (right axis) for all patients receiving irinotecan‐based treatment (left) or for patients treated with irinotecan as single agent (right).

Given the high frequency of increased TOPO1 expression in this cohort of patients, a sub‐analysis was conducted to explore the efficacy of irinotecan on lesions presenting with an overexpression of the TOPO1. For this analysis, data from the previously published Side Out 1 trial and Side Out 2 trial were pulled and concomitantly evaluated [13]. For both cohorts, TOPO1 expression was measured by the same CAP/CLIA accredited laboratory and examples of the IHC scoring system are shown in Fig. S2. Across the two cohorts, a total of 23 patients with TOPO1 overexpression were treated with irinotecan in combination (*n* = 12) or as single agent (*n* = 11) (Table S6). Best overall response in this group with irinotecan‐based therapy was available for 22 of the 23 patients, including two partial response (9.1%) and 14 stable disease (63.6%) for a combined clinical benefit in 72.7% (16/22). GMI data were available for 21 of the 23 patients; 14/21 (66.7%) patients met or exceeded a GMI of 1.3; in the single agent group, 6/11 (55%) patients met the primary objective (Fig. 5). Taken together, these data indicate that the multi‐omic approach proposed is uniquely suited for identifying emerging biomarkers and assessing their frequencies across subgroups of cancer patients. This information can serve as a groundwork for designing hypothesis‐driven analyses to explore the predictive and therapeutic roles of these biomarkers in future larger scale studies.

## 4 Discussion

Given the high vulnerability and mortality rate associated with metastatic progression, especially in heavily pretreated patients, unwinding the biological complexity of the disease and defining effective therapeutic and diagnostic gold standards remains a major priority for the breast cancer community. Routine testing of the efficacy of chemotherapy agents typically does extend beyond 2–3 lines of therapy. Thus, the best treatment is largely unknown after 2 lines of therapy for this patient population. Therefore, guidance on optimal therapy is desperately needed for patients with progressive disease. The utilization of biomarker‐enriched data for patients’ stratification to treatment has become the pillar of precision oncology globally [17]. In 2017, over 30% of industry‐sponsored trials utilized biomarker‐driven treatment selections, and however, the predominant use of genomic sequencing alone limits the number of patients for which precision oncology is a viable option [5, 6, 7, 18, 19, 20].

In this work, we demonstrated the unique role of comprehensive MoMP that combines targeted NGS, protein, and phosphoprotein analysis in helping a molecular TSC select treatment for heavily pretreated MBC patients affected by refractory disease with PFS < 6 months. A unique aspect of the trial was the collection of mulit‐omic data including NGS along with IHC and RPPA‐based data s to identify genomic‐dependent and independent signaling events modulated by the tumor ecology. Targetable alterations and/or chemo‐predictive markers from our expanded analysis were identified in 92% of patients enrolled in the study, a significantly higher actionability rate than seen in recent genomics‐only‐based trials [5]. Data were delivered to the molecular tumor board in a time frame highly compatible with clinical practice. In line with previously published data, more than 50% of patients enrolled in the trial benefited from this approach [12, 13]. Efficacy of treatments informed on the basis of individual MoMP should be further compared to empirical physician choice in randomized prospective clinical trials where GMI data are collected alongside other primary end points of response.

The development of a publicly available relational database, where MoMP are collected along with patients’ medical histories and outcome data, is another unique outcome of this work. While numerous online platforms capturing molecular information of primary tumors are available to the breast cancer community, molecular information from metastatic lesions is sparse and fragmented. The relational database developed as part of this study provides access to a wide range of information collected through the Side Out‐sponsored trials [15] as well as an open data‐sharing platform for the scientific community. This database may provide opportunities for retrospective pooled biomarker analyses and for identifying targets frequently deregulated in metastatic lesions.

As an example, the pooled analysis of the Side Out 1 and 2 trials identified TOPO1 as a protein frequently overexpressed in MBC and as a potential predictor of response to irinotecan‐based treatment, as previously described [21, 22, 23, 24, 25]. Irinotecan as a single agent, when given after an anthracycline or taxane, has an objective response rate of 14% when given every 3 weeks and 23% when given weekly [26]. However, in our population, which was substantially more heavily pretreated, irinotecan‐based therapies produced a lower objective response rate (9.1%) along with a high rate of stable disease for an overall clinical benefit of 73%. A systematic review exploring clinical efficacy of irinotecan in MBC has shown response rate between 5–23% and 14–64%, respectively, for single agent irinotecan or in combination with other chemotherapeutic compounds [27]. To our knowledge, there is no clinical data published on response rates to irinotecan‐based treatments in MBC patients with an average of 4 prior lines of therapy. In our pooled analysis, 67% of patients receiving irinotecan in combination and 55% of patients treated with irinotecan as single agent reached a GMI ≥ 1.3, which seems to exceed previously reported results in unselected populations [26]. However, comparing these results with previous published analyses is out of the scope of this work and challenging due to the high number of previous treatments of our study cohorts and to the heterogeneous ER/PR/HER2 profile of our patients.

While this work provides encouraging observations on the role of MoMP as a tool that provides physicians an expanded rationale for molecularly informed treatment selection for MBC patients, a few limitations need to be addressed. First, the GMI remains an exploratory tool for pilot analyses in heavily pretreated breast cancer patients and does not necessarily correlate with other response indicators. However, for this specific analysis, we have selected this outcome measurement for the following reasons. First, this work was designed as the continuation of our previous efforts to analyze the role of MoMP in treatment selection for patients with advance metastatic cancers [12, 13]. Thus, the selection to keep the GMI as our primary outcome measurement. As previously reported, TTP in metastatic patients usually decreases in sequential line of treatment. As recently reported by Italiano *et al*. [28], a GMI greater than 1 is an indicator of a positive response to treatment given the natural progression of the disease. Thus, an increase in TTP of 30%, although arbitrary, should be considered an unexpected and positive result, as previously indicated [29]. In addition, the selection of GMI as the main outcome measure was also driven by the study design and objectives. Because the primary objective of the trial was to validate the role of MoMP in treatment selection using a single‐arm design and our cohort was highly heterogeneous, finding a historic control is extremely challenging.

In a pilot analysis conducted by Von Hoff *et al*. assessing the role of molecular profiling across metastatic tumors using GMI as the main outcome measure, a GMI > 1.3 was detected in 27% of patients with rates ranging between 44% and 20% based on the type of tumors analyzed [12]. In this patient population with an average of 3.94 lines of previous treatments, of which 60% (15/25) had already received 3 or more previous lines of therapy and 32% (8/25) had received more than six lines of therapy, 28% of patients were without disease progression four months after treatment initiation. Best response was reported as stable disease (16) or partial response (3) in 76% of the 25 patients analyzed. Undoubtedly, these results exceed expected response rates in heavily pretreated and progressing MBC patients, suggesting that MoMP‐driven treatment selection warrants further investigations. Thus, the clinical efficacy of MoMP‐informed treatments needs to be evaluated in prospective studies where response to treatment is directly compared to physician’s choice in a randomized setting.

Lastly, while our pilot study aimed to determine whether MoMP could aid treatment decisions in heavily pretreated patients, a significant limitation of our work was the inability to effectively match each patient to the treatment rationalized by the biomarker landscape due to difficulties in obtaining low‐cost therapeutic options. Nevertheless, we were able to demonstrate some potential biomarker‐therapy signals that will require further validation such as the TOPO1‐ironotecan finding.

## 5 Conclusions

Taken together, our data suggest that the efficacy of MoMP‐informed treatment selection for FDA‐approved regimens in refractory heavily pretreated MBCs merits further evaluation in a prospective randomized setting. The potential of MoMP, combining NGS, proteins, and phosphoproteins data capturing activation levels of FDA‐approved drug targets, should be further tested with experimental agents to broaden treatment selection and to evaluate its role in early phases of the drug discovery and clinical testing. Finally, dynamic MoMP coupled with longitudinal tumor biopsy sample collections may provide a novel means for identifying targetable molecular events associated with clonal evolution, resistance to treatment, and dynamic adaptation to the host microenvironment.

## Conflict of interest

The authors are inventors on US Government and University assigned patents and patent applications that cover aspects of the technologies discussed such as Laser Capture Microdissection and Reverse Phase Protein Microarrays. As inventors, they are entitled to receive royalties as provided by US Law and George Mason University policy. MP, LL, JW, and EP receive royalties from TheraLink Technologies, Inc. MP, LL, and EP are consultants to and/or shareholders of TheraLink Technologies, Inc; EP is shareholder and consultant of Perthera, Inc. SPA and NJR served as consultants of Paradigm Diagnostics. SPA is employed by Newave Pharmaceutical Inc, and is a consultant for SDPO and Exact Sciences.

### Peer Review

The peer review history for this article is available at https://publons.com/publon/10.1002/1878‐0261.13091.

## Author contributions

DWN, MJ, NJR, and SPA treating physicians; AC, ACK, DA, DML, DWC, EP, JA, JDC, JDW, KAH, LL, MP, RIG, and SW molecular profiling; AB data analysis and visualization; EB, SA, JW, FA, and MP Database construction and data entry; EP, NJR, and SPA trial design; LV and BD trial manage and administrative support.

## Supporting information


**Table. S1**. List of inclusion and exclusion criteria for the Side Out 2 trial.
**Table S2**. Biomarkers measured as part of the MoMP for patients enrolled in the Side Out 2 trial
**Table S3**. Summary of treatment selected by physicians empirically compared to regimens selected based on MoMPs.
**Table S4**. Summary of adverse events recorded during the Side Out 2 trial.
**Table S5**. Summary of Best Response for the 25 patients evaluated in the trial (PD: progressive disease, SD: stable disease; PR: partial response, respectively).
**Table S6**. Regimen, GMI, and overall best response for the 22 patients with TOPO1 overexpression that were treated with an irinotecan‐based regimen.
**Fig. S1**. GMI values of the 25 patients enrolled in the Side Out 2 trial for whom treatment was selected based on MoMP.
**Fig. S2**. Selected examples of TOPO1 staining in two metastatic breast cancer patients.

## Data Availability

All clinical and molecular information described in the manuscript can be accessed through the MBC bioinformatic portal at https://sideoutfoundation.gmu.edu/.
