# Supplementary material for: Multi‐omic molecular profiling guide’s efficacious treatment selection in refractory metastatic breast cancer: a prospective phase II clinical trial
Source: Mol Oncol. 2021 Sep 12;16(1):104–15. doi: 10.1002/1878-0261.13091 (PMC8732340; doi:10.1002/1878-0261.13091)
Supplement: Supplementary file 1 — Table. S1. List of inclusion and exclusion criteria for the Side Out 2 trial. Table S2. Biomarkers measured as part of the MoMP for patients enrolled in the Side Out 2 trial Table S3. Summary of treatment selected by physicians empirically compared to regimens selected based on MoMPs. Table S4. Summary of adverse events recorded during the Side Out 2 trial. Table S5. Summary of Best Response for the 25 patients evaluated in the trial (PD: progressive disease, SD: stable disease; PR: partial response, respectively). Table S6. Regimen, GMI, and overall best response for the 22 patients with TOPO1 overexpression that were treated with an irinotecan‐based regimen. Fig. S1. GMI values of the 25 patients enrolled in the Side Out 2 trial for whom treatment was selected based on MoMP. Fig. S2. Selected examples of TOPO1 staining in two metastatic breast cancer patients. [file MOL2-16-104-s001.docx]

**Supplementary Table S1.** List of inclusion and exclusion criteria for the Side Out 2 trial.

| **Inclusion Criteria** |
| --- |
| Have recurrent disease with < 6 months of treatment under the last therapy |
| Understand and provide written informed consent and HIPAA Authorization prior to initiation of any study-specific procedures |
| Have a life expectancy > 3 months |
| Have a diagnosis of metastatic breast cancer with measurable disease (RECIST 1.1) |
| Have progressed on ≥ 1 prior chemotherapeutic and/or hormonal regimen for advanced disease |
| Have documented progression (by RECIST 1.1) on the treatment regimen immediately prior to entering this study |
| Be ≥ 18 years of age |
| Have a ECOG score of 0-1 |
| Have been off their prior regimen for ≥ 3 weeks or 5 x half-life of drug |
| Have adequate organ and bone marrow function assessed by: CBC, differential, platelet count, hemoglobin, ALT/SGPT, AST/SGOT, AP, total bilirubin, LDH, creatinine clearance or creatinine, albumin, PT, APTT |
| Have a negative pregnancy test and agree to use contraception during the study and for at least one month after treatment discontinuation when applicable |
|  |
| **Exclusion Criteria** |
| Have a tumor biopsy intended for use in the current study that was performed more than 2 months prior to analysis |
| Have metastatic lesion that is not accessible to biopsy |
| Had > 6 months treatment under the last line of therapy |
| Have interventional cancer therapy conducted after the biopsy was collected prior to analysis |
| Have symptomatic CNS metastasis |
| Have any previous history of another malignancy within 5 years of study entry |
| Have uncontrolled concurrent illness including, active serious infection, symptomatic congestive heart failure, unstable angina pectoris, unstable cardiac arrhythmias, psychiatric illness |
| Have known HIV, HBV, HCV infection |
| Are pregnant, breast-feeding or unwilling to use adequate contraception when applicable |

**Supplementary Table S2.** Biomarkers measured as part of the MoMP for patients enrolled in the Side Out 2 trial.

Panel A

| **Protein Expression by IHC** |
| --- |
| Androgen Receptor (AR) |
| Estrogen Receptor (ER) |
| Progesterone Receptor (PR) |
| Secreted Protein Acidic and Rich in Cysteine (SPARC) |
| DNA Topoisomerase II Alpha (TOP2A) |
| DNA Topoisomerase 1 (TOPO1) |
| Thymidylate Synthase (TS) |

Panel B

| **mRNA Expression** | | | **DNA Mutation** | | |
| --- | --- | --- | --- | --- | --- |
| AR | ERBB3 | PTEN | ABCB1 | EZH2 | MET |
| AREG | ERCC1 | PTGS2 | AKT1 | FGD4 | MSH6 |
| ARID1A | EREG | PTPN6 | ALK | FGFR2 | MSI |
| BAD | ESR1 | RELA | BRAF | FGFR3 | MTOR |
| BAX | EZH2 | RPS6KB1 | BRCA1 | FLT3 | NRAS |
| BCL2 | FGFR1 | RRM1 | BRCA2 | GATA3 | PDGFRA |
| BIRC5 | IGF1R | SLC29A1 | CHEK1 | GNAQ | PIK3CA |
| BRCA1 | KDR | SSTR2 | CREBBP | GNAS | PTCH1 |
| CA9 | KIT | TNF5F13 | CSF1R | HRAS | RET |
| CDA | LRP6 | TOP2A | DOR2 | IDH1 | ROS1 |
| CDH1 | MET | TUBB3 | EGFR | IDH2 | SMO |
| CES2 | MGMT | TYMP | EP300 | IGF1R | STAT3 |
| CHUK | MITF | TYMS | EPHAS | KIT | TGFBR2 |
| DCK | MTOR | VEGFA | ERBB2 | KRAS | TP52 |
| DHFR | NFKB1 |  | ERBB4 | MAP2K1 | TSC1 |
| DPYD | PARP1 |  | ERCC2 | MAP2K2 | TSC2 |
| EPHA2 | PDGFRB |  | ERRFI1 | MAP3K1 |  |
| ERBB2 | PGR |  | ESR1 | MDM2 |  |
| **Copy Number Variation** | | **Protein Expression by IHC** | | **Fusion Panel** | |
| 19Q | ERBB2 | ALK | RET | ALK | RAF1 |
| 1P | FGF3 | AR | ROS1 | AXL | RET |
| ALK | FGF4 | CAIX | TOPO1 | BRAF | ROS1 |
| AURKA | FGFR1 | ER | TP | CCND1 | THADA |
| C11ORF30 | FGFR2 | HENT1 | TRKpan | FGFR1 |  |
| CCND1 | FGFR3 | HER2 | TS | FGFR2 |  |
| CCND2 | MET | IDO | TUBB3 | FGFR3 |  |
| CCND3 | MYC | MET | MLH1 | MET |  |
| CCNE1 | MYCN | MGMT | MSH2 | NRG1 |  |
| CDK4 | NTRK1 | PD1 | MSH6 | NTRK1 |  |
| CDK6 | SMAD4 | PDL1 | PMS2 | NTRK2 |  |
| CDKN2A | TOP2A | PR |  | NTRK3 |  |
| EGFR | VEGFA | PTEN |  | PPARG |  |

Panel C

| **Protein Activation Mapping by RPPA** |
| --- |
| pAKT S473 |
| pc-Abl Y735 |
| pEGFR Y1068 |
| pHER2 Y1248 |
| pHER3 Y1289 |
| pERK 1/2 T202/Y204 |
| pp70S6K T389 |
| pPDGFR Y751 |
| PTEN |
| pRet Y905 |
| pSrc Y527 |

**Supplementary Table S3.** Summary of treatment selected by physicians empirically compared to regimens selected based on MoMPs. In bold, samples for which physician choice matched, at least partially, the treatment selected after the molecular profile was performed.

| Subject ID | GMI | Physician choice | MoMP-based selected treatment |
| --- | --- | --- | --- |
| 02-027 | 0.3 | Everolimus; Exemestane | Irinotecan; Megestrol Acetate |
| 02-012 | 0.4 | Gemcitabine | Capecitabine; Irinotecan; Megestrol Acetate |
| 02-037 | 0.5 | Gemcitabine | Irinotecan |
| ***02-043**** | ***0.5*** | ***Eribulin*** | ***Eribulin*** |
| 02-008 | 0.5 | Eribulin | Everolimus; Exemestane |
| 02-006 | 0.6 | Gemcitabine | Capecitabine; Lapatinib |
| 02-007 | 0.7 | Doxorubicine | Eribulin; Irinotecan; Lapatinib; Letrozole |
| 02-021 | 0.8 | Nab-paclitaxel | Everolimus; Exemestane |
| 02-032 | 0.8 | Capecitabine | Eribulin |
| ***02-018**** | ***0.9*** | ***Capecitabine*** | ***Capecitabine*** |
| 02-041 | 1.2 | Paclitaxel | Irinotecan |
| 02-020 | 1.3 | Capecitabine | Everolimus; Exemestane |
| ***02-023**** | ***1.4*** | ***Capecitabine*** | ***Capecitabine****;* Irinotecan; Paclitaxel |
| 02-039 | 1.4 | Paclitaxel | Irinotecan; Trastuzumab |
| 02-014 | 1.4 | Eribulin | Irinotecan |
| 02-025 | 1.8 | Everolimus; Exemestane | Capecitabine |
| 02-009 | 2.2 | Eribulin | Capecitabine; Megestrol Acetate; Vinorelbine |
| 02-003 | 2.4 | Capecitabine | Paclitaxel |
| 02-017 | 2.8 | Trastuzumab; Vinorelbine | Capecitabine; Lapatinib |
| 02-029 | 3.8 | Nab-paclitaxel | Irinotecan |
| ***02-036**** | ***4.2*** | ***Capecitabine*** | ***Capecitabine;*** Irinotecan |
| 02-010 | 6.1 | Doxorubicine | Irinotecan |
| ***02-019**** | ***7.2*** | ***Pertuzumab-containing regimen*** | Docetaxel; ***Pertuzumab***; Trastuzumab |
| 02-011 | 8.5 | Capecitabine | Capecitabine; Irinotecan |
| 02-028 | 15.9 | Nab-paclitaxel | Capecitabine |

**Supplementary Table S4.** Summary of adverse events recorded during the Side Out 2 trial.

| **Grade 1-2 adverse events** |
| --- |
| Gastrointestinal symptoms (n=2) |
| Skin lesions (n=2) |
| Upper respiratory infection (n=1) |
| Neutropenia (n=1) |
| Urinary tract infections (n=2) |
| Neutropenia and one urinary tract infection required delay or treatment adjustment. |
| **Grade 3 adverse events** |
| Febrile neutropenia (n=2) |
| Leg pain (n=1) |
| Gastrointestinal symptoms (n=2) |
| Three of the grade 3 adverse events were classified as severe and the two febrile neutropenia led to delay or treatment interruption. |
| **Grade** **4 adverse events** |
| Severe neutropenia (n=1) |
| Pneumonitis (n=1) |
| Pneumonitis affected treatment administration |

**Supplementary Table S5.** Summary of Best Response for the 25 patients evaluated in the trial (PD: progressive disease, SD: stable disease; PR: partial response, respectively).

| Subject ID | GMI | Best response |
| --- | --- | --- |
| 02-027 | 0.3 | PD |
| 02-012 | 0.4 | SD |
| 02-037 | 0.5 | PD |
| 02-043 | 0.5 | NA |
| 02-008 | 0.5 | PD |
| 02-006 | 0.6 | SD |
| 02-007 | 0.7 | SD |
| 02-021 | 0.8 | SD |
| 02-032 | 0.8 | PD |
| 02-018 | 0.9 | PD |
| 02-041 | 1.2 | SD |
| 02-020 | 1.3 | SD |
| 02-023 | 1.4 | SD |
| 02-039 | 1.4 | SD |
| 02-014 | 1.4 | SD |
| 02-025 | 1.8 | PR |
| 02-009 | 2.2 | SD |
| 02-003 | 2.4 | SD |
| 02-017 | 2.8 | SD |
| 02-029 | 3.8 | SD |
| 02-036 | 4.2 | SD |
| 02-010 | 6.1 | SD |
| 02-019 | 7.2 | SD |
| 02-011 | 8.5 | PR |
| 02-028 | 15.9 | PR |

**Supplementary Table S6.** Regimen, GMI, and overall best response for the 22 patients with TOPO1 overexpression that were treated with an irinotecan-based regimen.

| Subject ID | Treatment | GMI | Best Response |
| --- | --- | --- | --- |
| 01-101 | Irinotecan | 1.977 | SD |
| 01-105 | Irinotecan | 0.319 | PD |
| 01-107 | FOLFIRI | 1.303 | SD |
| 01-109 | Irinotecan + Trastuzumab | 7.156 | SD |
| 01-110 | FOLFIRI | 6.873 | SD |
| 01-112 | FOLFIRI | 0.046 | PD |
| 01-113 | Irinotecan + Exemestane | 2.260* | SD |
| 01-115 | Irinotecan + Fluorouracil | 1.684 | SD |
| 01-117 | Irinotecan | 3.408 | SD |
| 01-118 | FOLFIRI | 2.527 | PR |
| 02-007 | Irinotecan | 0.851 | PD |
| 02-010 | Irinotecan | 3.843 | SD |
| 02-011 | Irinotecan + Capecitabine | 8.539 | PR |
| 02-012 | Irinotecan + Capecitabine | 0.366 | PD |
| 02-014 | Irinotecan | 1.373 | SD |
| 02-023 | Irinotecan + Capecitabine | 1.37 | SD |
| 02-027 | Irinotecan | 0.429 | PD |
| 02-029 | Irinotecan | 3.512 | SD |
| 02-037 | Irinotecan | 1.145 | PD |
| 02-036 | Irinotecan | 2.139 | SD |
| 02-039 | Irinotecan + Trastuzumab | 1.429 | SD |
| 02-041 | Irinotecan | 1.166 | SD |

* Images were not collected within the GMI window.

**Supplementary Figure S1.** GMI values of the 25 patients enrolled in the Side Out 2 trial for whom treatment was selected based on MoMP.

**Supplementary Figure S2.** Selected examples of TOPO1 staining in two metastatic breast cancer patients. Panel A and C Hematoxylin and Eosin of the tissue specimens; Panel B, positive staining for TOPO1; and Panel D, negative staining for TOPO1.
